# Graph Theory Analysis of the Cortical Functional Network During Sleep in Patients With Depression

**DOI:** 10.3389/fphys.2022.858739

**Published:** 2022-05-27

**Authors:** Yingjie Song, Kejie Wang, Yu Wei, Yongpeng Zhu, Jinfeng Wen, Yuxi Luo

**Affiliations:** ^1^ School of Biomedical Engineering, Sun Yat-sen University, Guangzhou, China; ^2^ Department of Psychology, Guangdong, 999 Brain Hospital, Guangzhou, China; ^3^ Guangdong Provincial Key Laboratory of Sensing Technology and Biomedical Instruments, Sun Yat-sen University, Guangzhou, China

**Keywords:** depression, sleep, electroencephalography, functional connectivity, graph theory

## Abstract

Depression, a common mental illness that seriously affects the psychological health of patients, is also thought to be associated with abnormal brain functional connectivity. This study aimed to explore the differences in the sleep-state functional network topology in depressed patients. A total of 25 healthy participants and 26 depressed patients underwent overnight 16-channel electroencephalography (EEG) examination. The cortical networks were constructed by using functional connectivity metrics of participants based on the weighted phase lag index (WPLI) between the EEG signals. The results indicated that depressed patients exhibited higher global efficiency and node strength than healthy participants. Furthermore, the depressed group indicated right-lateralization in the δ band. The top 30% of connectivity in both groups were shown in undirected connectivity graphs, revealing the distinct link patterns between the depressed and control groups. Links between the hemispheres were noted in the patient group, while the links in the control group were only observed within each hemisphere, and there were many long-range links inside the hemisphere. The altered sleep-state functional network topology in depressed patients may provide clues for a better understanding of the depression pathology. Overall, functional network topology may become a powerful tool for the diagnosis of depression.

## Introduction

Depression, with severe symptoms affecting the life of sufferers, is considered to be one of the most serious and common disorders in the modern world (World Health Organization, 2019). Considering the psychological burden of depression on patients and the economic impact on the society, the neurophysiological research on depression is imperative. However, multiple causes of depression and their interactions make accurate pathology studies difficult (Hou et al., 2016; Peng et al., 2019; Zhang et al., 2019). Notably, depressed patients have abnormal brain activity during sleep (Steiger and Pawlowski, 2019), making it a breakthrough in the diagnosis of depression.

Sleep plays an important role in the process of psychiatric comorbidity (Hou et al., 2016; Leitgeb et al., 2020). Sleep performance in patients with certain psychiatric disorders, including depression, can, to some extent, predict the onset state and future treatment outcomes (Tononi and Cirelli, 2006; Hou et al., 2016). It has been reported that depressed patients showed a decrease in the total sleep time, sleep efficiency, slow-wave sleep during NREM, and REM sleep latency and an increase in the REM cycle density and duration (Armitage, 2007; Hein et al., 2019; Riemann et al., 2020). As the brain’s response to stimuli diminishes during sleep, the characteristics of some neurological diseases are more easily observed (Clarke and Harvey, 2012; Richardson and Adams, 2018). Relevant studies have indicated that there are abnormal changes in the sleep brain network in depressed patients. Based on the synchronous likelihood method, the global synchronization of depressed patients has been confirmed to be lower in the δ, θ, and σ bands in the sleep state (Leistedt et al., 2009; Hasanzadeh et al., 2020). In addition, the recent study in our group successfully applied sleep electroencephalography (EEG) functional connectivity to detect depressed patients, and the obtained results were delightful (Lian et al., 2021).

Functional connectivity (FC), a neuroscience tool based on the mathematical framework, describes the temporal correlations between the brain regions (Lv et al., 2015; Desjardins et al., 2016; Huang et al., 2016). Furthermore, dynamic functional connectivity (dFC), which introduces the time dimension to provide dynamic information, has recently been applied to estimate the cortical activities in the task state (Eken et al., 2019; Ding et al., 2020; Li R. et al., 2020). However, to the best of our knowledge, insufficient knowledge is available on the dynamic characteristics of FC during sleep. The analysis of dFC with a focus on the switching periods of sleep stages may provide some new clues about the cortical activity differences in depressed patients during sleep.

Currently, graph theory is a method that uses FC to characterize topological features based on EEG. Through graph theory, complex brain systems can be abstracted into simple geometric representations, emphasizing the use of network topology to simulate the fragility and elasticity of the brain and reducing the complexity of analytical processes (i.e., many nodes and the relationships between them) (Rubinov and Sporns, 2010). Studies have pointed out that graph theory is a reliable method to extract brain connectivity based on the EEG signals to investigate the physiological impact of diseases (Heckmann et al., 2015; Hasanzadeh et al., 2020; Majeed and Rauf, 2020). Additionally, it has the potential to be an effective descriptive tool for predicting diseases, significantly identifying quantitative and specific biomarkers, and understanding brain dysfunction from the structure and morphology (Heckmann et al., 2015; Matyi et al., 2021; Drange et al., 2022). Our recent study suggested that the weighted phase lag index (WPLI) between the EEG signals well-characterized the abnormal FC in depressed patients, for which we suppose that the graph theory based on WPLI might be a powerful tool for detailing the abnormal cortical activities in depressed patients.

The aim of the present work was to construct cortical networks based on the WPLI among the sleep EEG signals and to compare graph metrics in depressed patients and healthy participants. Through the description of cortical networks, it is hoped to build a bridge between the current neurological research and the performance of the abnormal cortical activities in depressed patients.

**FIGURE 1:**
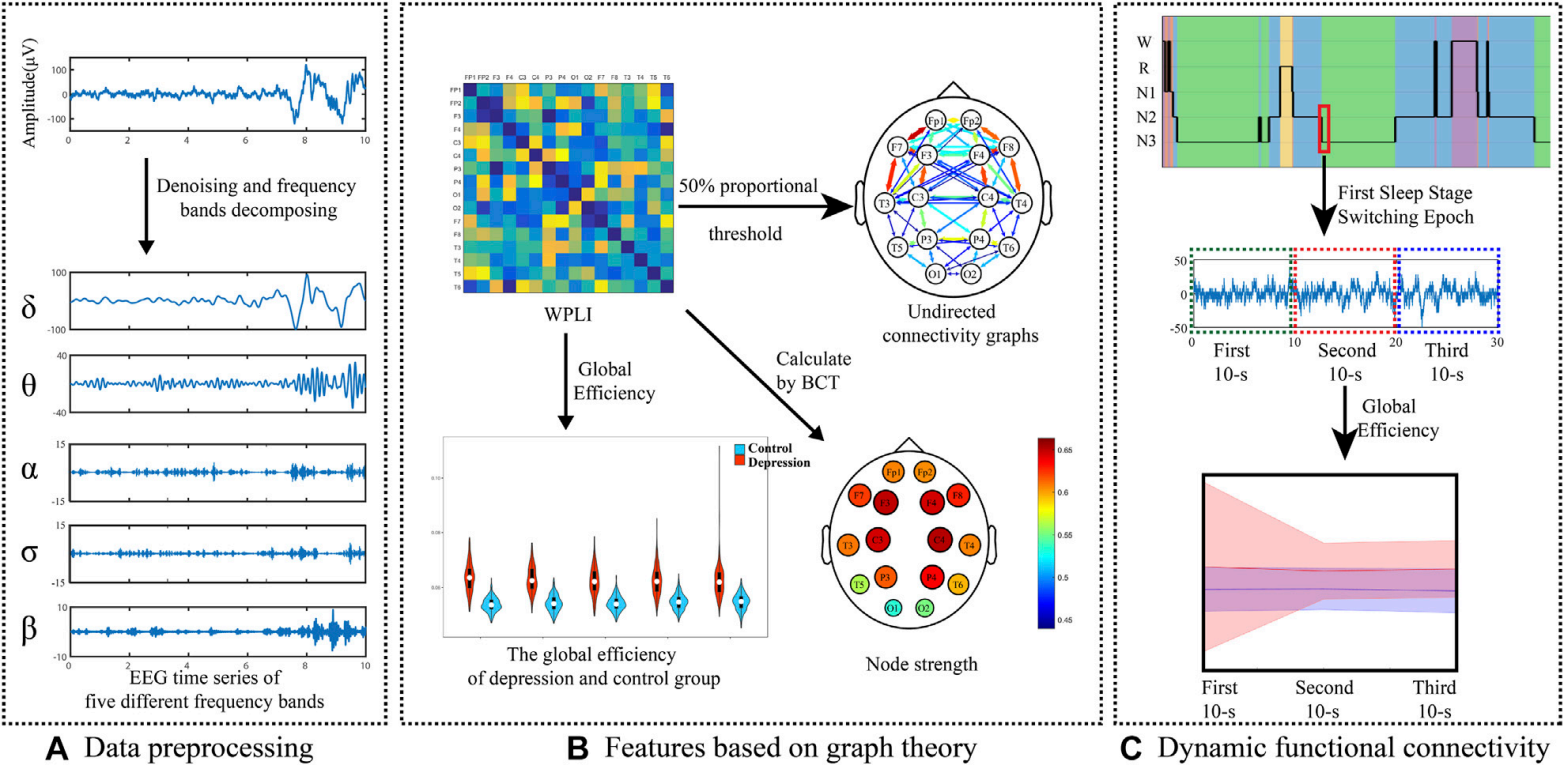
Framework of this study.

The rest of the manuscript is structured as follows: in the Methods section, we describe the participants, EEG data recording, and preprocessing. After completing the analysis, the results of the experimental data are presented and discussed in the Results section and Discussion section. Finally, Conclusions is included in the manuscript. Figure 1 shows the framework of this study.

## Materials and Methods

### Participants

In this study, 51 subjects (25 healthy participants and 26 depressed patients) were enrolled. The patients were recruited from Guangdong 999 Brain Hospital and were diagnosed by a psychiatrist, according to the Diagnostic and Statistical Manual of Mental Disorders, Fourth Edition (DSM-IV). The participants with depression were evaluated using the Hamilton Depression Scale (HAMD) and the Self-Rating Depression Scale (SDS). Depressed patients with drug abuse, suicide risk, pregnancy, current or history of head injuries, seizures, or epilepsy were excluded. All the healthy participants (approximately 20 years old) were undergraduates at Sun Yat-Sen University and were free from sleep disorders such as insomnia, depression, and sleep apnea. The male/female in the control and depression groups was 11/14 and 15/11, respectively. All the participants were accustomed to using the right hand. All the procedures were performed in compliance with the 1964 Helsinki declaration and its subsequent amendments. This study was approved by the Ethics Committee of Guangdong 999 Brain Hospital (approval number: 2020-010-059), and the research was carried out here. The subjects were informed of the experimental procedures in advance and told not to drink caffeinated or alcoholic beverages and not to overeat for 24 h before arriving at the sleep center of the hospital. Informed consent was obtained from all the individual participants.

All the participants underwent overnight polysomnography (PSG) examination, which lasted for 9–10 h. Recordings were performed by using a Compumedics Profusion EEG Recording System with Neuvo Amplifier. PSG recordings included 16 EEG channels [regarding six brain areas: frontal poles (Fp1/M2 and Fp2/M1), frontal lobe (F3/M2, F4/M1, F7/M2, and F8/M1), central (C3/M2 and C4/M1), occipital lobes (O1/M2 and O2/M1), parietal lobes (P3/M2 and P4/M1), and temporal lobes (T3/M2, T4/M1, T5/M2, and T6/M1)] based on the standard 10–20 system, electrooculography (EOG), electrocardiography (ECG), electromyography (EMG), oral and nasal respiratory airflow, chest and abdomen breathing movement, blood oxygen saturation, snoring, leg movement, and body position. The sampling frequency of the EEG, EOG, and EMG signals was 500 Hz, while that of the respiration signal and oxygen saturation were set to 100 and 10 Hz, respectively. Sleep stages were scored by experienced sleep technicians, according to the AASM criteria as Wake, N1, N2, N3, and REM (Saper et al., 2010). The information of the subjects is shown in Table 1.

**TABLE 1:** Demographic features and polysomnographic parameters of the depressed group and controls.

Variable	Control	Depression	*p*-Value
Gender (male/female)	11/14	15/11	0.910
Age (years)	20.00 ± 1.50	21.60 ± 7.04	0.947
SDS score	40.92 ± 6.99	67.3 ± 9.91	<0.001
HAMD score	2.08 ± 1.57	25.5 ± 6.28	<0.001
Total record time	471.30 ± 52.45	585.05 ± 51.21	/
NREM1 time (min)	24.66 ± 12.88	22.81 ± 15.84	0.447
NREM2 time (min)	176.56 ± 38.86	249.38 ± 88.89	0.002
NREM3 time (min)	144.22 ± 36.97	150.36 ± 62.14	0.766
REM time (min)	81.12 ± 20.70	102.67 ± 47.55	0.270
Total sleep time (min)	426.56 ± 42.04	525.21 ± 71.65	/

The results were expressed as the mean ± standard deviation, except for gender. The chi-squared test was used for the comparison of gender, and the other comparisons were assessed using the Mann–Whitney U test.

HAMD, Hamilton Depression Scale; SDS, Self-rating Depression Scale.

### EEG Signal Preprocessing

Segments containing obvious artifacts were removed by visual inspection. A total of 25194/19150 segments of depressed patients/healthy participants were obtained (1467/567 W epochs, 4692/2945 R epochs, 1030/545 N1 epochs, 11608/8138 N2 epochs, and 6397/6955 N3 epochs).

The dFC analysis focused on the sleep stages’ switching periods. The first 30-s epochs at the beginning of each sleep stage were calculated. These 30-s segments were divided into three 10-s segments for calculating the dFC. In total, 2739/1936 segments of depressed patients/healthy participants were obtained for the dFC analysis (313/202 W epochs, 164/258 R epochs, 427/442 N1 epochs, 1169/774 N2 epochs, and 666/260 N3 epochs).

A fourth-order zero phase shift Butterworth bandpass filter with a passband of 0.5–50 Hz was applied for signal preprocessing. The EEG data were estimated and processed in a moving window of 3 s (with an overlap of 2 s).

### Phase Synchronization Estimation

The analysis was performed in the five frequency bands of δ (0.5–4 Hz), θ (4–8 Hz), α (8–12 Hz), σ (12–16 Hz), and β (16–32 Hz).

The WPLI, explained by Vinck *et al.* and improved by Liao *et al.*, was calculated to measure the phase synchronization index for characterizing the FC (Liao et al., 2019). The WPLI introduces the imaginary part of the cross power spectrum to weigh the signal for improving the immunity to noise sources; thus, it can reflect the cortical oscillation relationship between different parts of the same brain area or between different brain areas. The WPLI is defined as follows:

$$\mathrm{WPL}I_{i,j,\tau }=\frac{\left|E\left\{\sin\left(\Delta \varphi_{\mathrm{i,j,\tau}}\right)\right\}\right|}{E\left\{\left|\sin\left(\Delta \varphi_{i,\mathrm{j,\tau}}\right)\right|\right\}},$$

where 0 $\leq WPLI_{i,j,\tau }\leq$ 1, and E () is the expected value operator. $\Delta \varphi_{i,j,\tau }$ , representing the phase difference between a pair of nodes i and j, was calculated using the formula

$$\Delta \varphi_{i,j,\tau }=\varphi_{i}\left(\tau \right)-\varphi_{j}\left(\tau \right).$$

The instantaneous phase φ(τ) is extracted by using a combined Morlet wavelet, which has a stable passband and a narrow transition band. A single Morlet complex wavelet is defined as follows:

$$\varphi \left(t\right)=\frac{1}{\sqrt{\pi f_{b}}}e^{2\mathrm{i\pi t}f_{c}}e^{-\frac{t^{2}}{f_{b}}},$$

where $f_{c}$ is the central frequency of the wavelet, and $f_{b}$ is the bandwidth parameter. The combined Morlet wavelet is obtained by superimposing multiple Morlet wavelets with different center frequencies $f_{M}$ . The central frequency of the Morlet wavelet is thus defined as follows:

$$f_{M}=f_{L}+M\times \Delta f,M=0\ldots N-1,$$

where Δf , which is the central frequency spacing of the wavelet, is 0.05 in this study. The corresponding $f_{L}$ and N are defined according to different frequency bands, such as $f_{L}=0.5$ and N = 70 for δ and $f_{L}=4$ N = 80 for θ. The Morlet combination is defined as the following formula:

$$\psi_{c}\left(t\right)=\frac{1}{C}\sum_{M=0}^{N-1}\sigma_{f_{M}}\left(t\right)=\frac{1}{C\sqrt{\pi f_{b}}}e^{-\frac{x^{2}}{f_{b}}}\sum_{M=0}^{N-1}e^{2\mathrm{i\pi}f_{M}t},$$

where C is the correction coefficient that makes the amplitude–frequency characteristic passband of the combined wavelet to be 1. The phase information within a fixed frequency band will be more accurate because of the high concentration of the Morlet wavelet in the time and frequency domains. The wavelet coefficient of the EEG signal S(t) , which is obtained from the electrode at time τ , is defined as follows:

$$W_{S}\left(\tau \right)=\int_{-\infty }^{+\infty }S\left(t\right)\psi_{C}^{*}\left(t-\tau \right)\mathrm{dt}=A\left(\tau \right)e^{i\varphi \left(\tau \right)}.$$

“∗” represents conjugation. The process and calculation formula of Morlet wavelet phase extraction can be obtained in the manuscript of Wang et al. (2011).

### Graph Theory

The weighted undirected network was based on the WPLI with rows and columns corresponding to two electrodes. To evaluate the characteristics of the weighted undirected network, some graph theory metrics were used by the Brain Connectivity Toolbox (BCT) (Rubinov and Sporns, 2010; Rossini, 2021). GE (global efficiency) and node strength were calculated to compare the functional networks in healthy participants and depressed patients. GE can be used to evaluate the separation and randomness of the cortical network. With respect to node strength, the sum of link weights around the node can exhibit the hub of the network. We built a binary network using a weighting matrix. According to previous research (Achard and Bullmore, 2007; Jalili, 2017; Kabbara et al., 2018), we fixed the density value at 0.30 for sparsity thresholding to construct a sparse matrix.

Rubinov and Sporns (2010) introduced the calculation formula and representative meaning of the parameters in graph theory. GE reflects exchanged efficient information in the network, and the measures quantified the network efficiency. GE is defined as follows:

$$E=\frac{1}{n}\sum_{i\in N}E_{i}=\frac{1}{n}\underset{i\in N}{\sum }\frac{\sum_{j\in N,j\neq i}d_{\mathrm{ij}}^{W-1}}{n-1}.$$

$E_{i}$ is the efficiency of node i. $d_{ij}$ is the distance between nodes i and j, and $d_{ij}$ is defined as

$$d_{\mathrm{ij}}^{W}=\;\sum_{a_{\mathrm{ij}}\in \mathrm{gi}↔j}^{W}f\left(W_{\mathrm{uv}}\right),$$

where f is a map from the weight to length, and gi↔j is the shortest weighted path between i and j. $W_{uv}$ is the link value in the 16∗16 matrix of the WPLI. In this study, we fixed the density value at 0.30 for sparsity thresholding to construct a sparse matrix. Consequently, when the WPLI > the threshold, the map f indicates that the data are not changed. When the WPLI < the threshold, the map f represents 0. The node strength, which can exhibit the hub of the network, is the sum of the link weights connected to the node in the weighted network. The node strength is defined as follows:

$$k_{i}^{W}=\sum_{j\in N,j\neq i}W_{\mathrm{ij}},$$

where $W_{ij}$ is the link weight between nodes i and j.

### Statistical Analysis

The statistical tests were carried out on five frequency bands in depressed patients and controls to access the cortical networks in depressed patients. The Kolmogorov–Smirnov test was used to verify the normality of the obtained data. Since GE, node strength, and WPLI did not satisfy the normal distribution or the variance homogeneity test, a nonparametric test (Mann–Whitney U test) was applied. To evaluate the laterality of the network, a paired T-test was used for the left and right hemispheres in the depressed patients and controls. The significance of the differences between the sleep stages for each frequency band was analyzed by the Kruskal–Wallis test followed by the *post hoc* Dunn–Bonferroni test. All the analyses were performed by IBM statistics software SPSS (version 22.0).

## Results

### Network Efficiency

**FIGURE 2:**
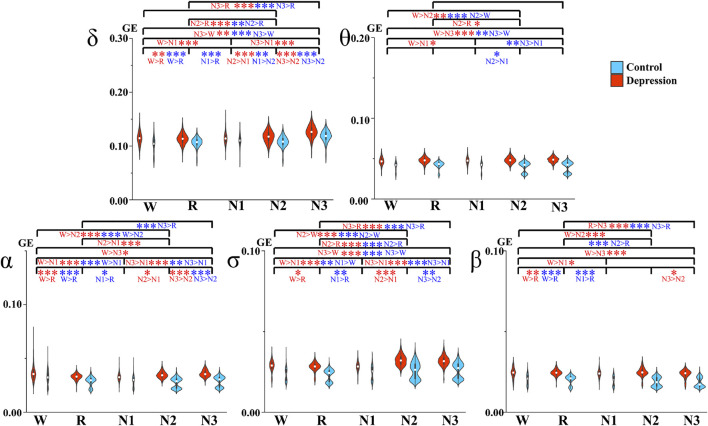
Average GE between different sleep stages. Red and blue asterisks denote the significant difference between two stages of the depression group and the control group, respectively. *p < 0.05, **p < 0.005, and ***p < 0.001 (Bonferroni correction).

The GE values and significant differences between the groups are shown in Figure 2. The depressed patients had significantly higher GE values than the controls in all the frequency bands and sleep stages studied. In addition, the differences between the patients and controls were greater than those between different sleep stages within each group.

**FIGURE 3:**
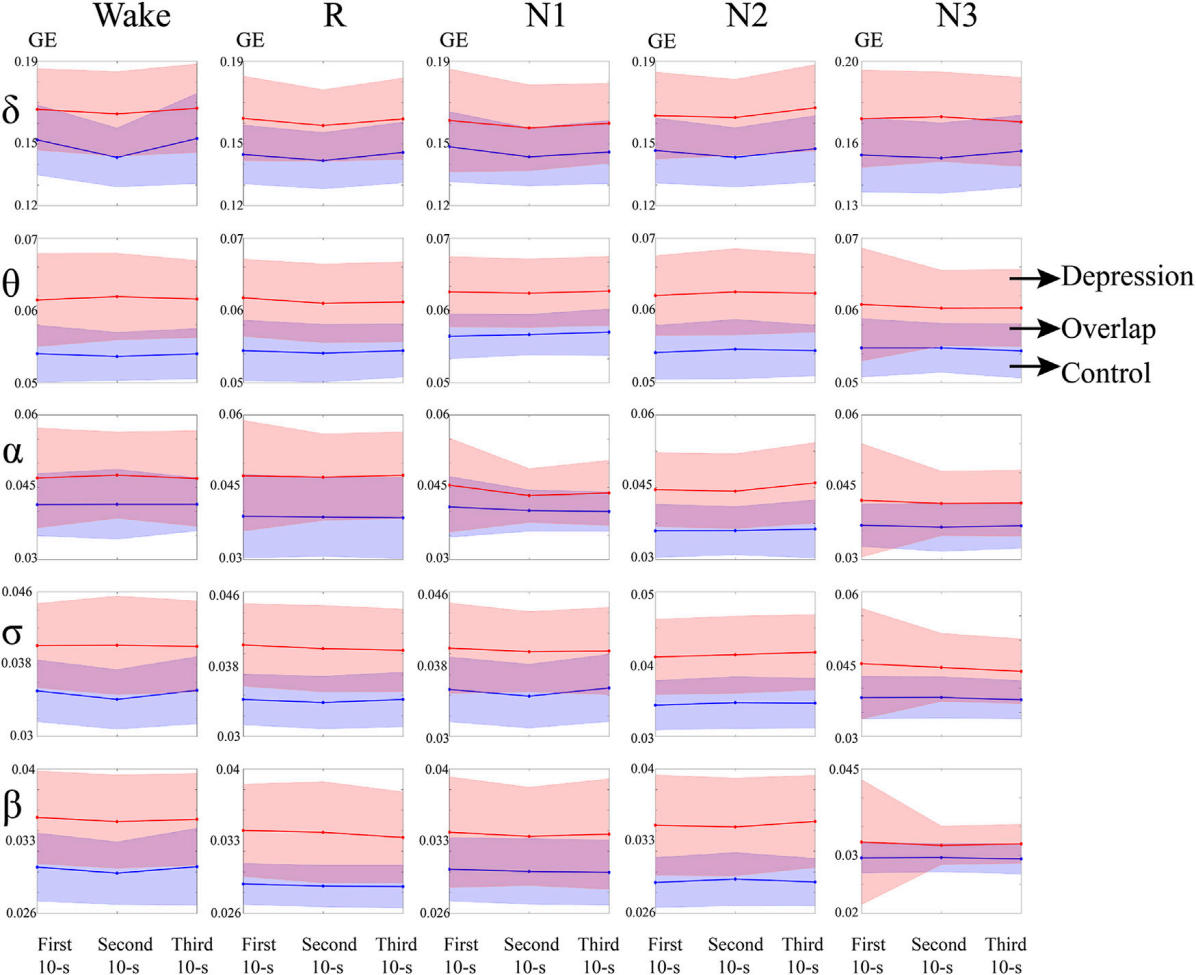
GE of the depressed and control groups during sleep switching. Red and blue represent the depressed group and control group, respectively. Purple is the overlapping part of the two groups.

Dynamic analysis of GE during switching of the sleep stages was also performed. The average global of the network and changes in the standard deviation of the two groups over these epochs are depicted in Figure 3. The standard deviation at the onset of the N3 stage became smaller in θ, α, σ, and β bands in the patient group, while that in the control group remained at a relatively low level.

Within the depressed and control groups, the differences across the sleep stages manifested differently in some frequency bands. We observed different results across Wake, N1, and REM between the groups. The depressed group had significant differences between Wake and N1 in all the researched bands, but the controls only exhibited a significant difference in the σ band, and this comparison result is opposite to that of the patients. However, the depressed group did not indicate any significant difference between REM and N1, but such differences were shown in most bands for the control group. With the deepening of N-REM sleep, which is from N1 to N2 and to N3, GE was increasing for both the groups in general, but the changes in θ and β bands were not so significant. Exceptions were as follows, in control: the GE of N1 was larger than N2 in the δ and β bands. Meanwhile, less significant differences were observed in θ for the two groups.

### Network Hubs

**FIGURE 4:**
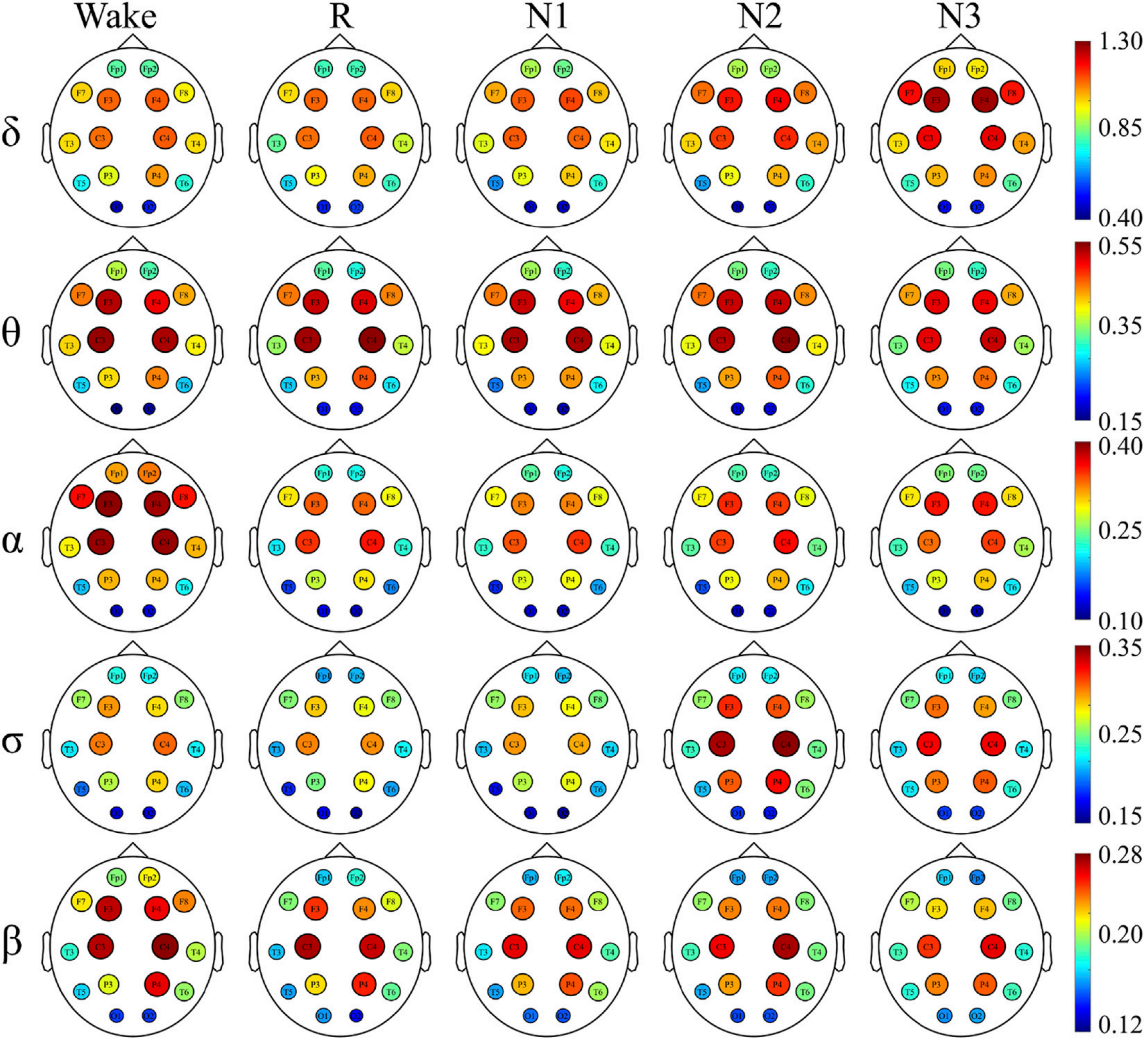
Node strength of the depressed group and red indicates a higher node strength.

**FIGURE 5:**
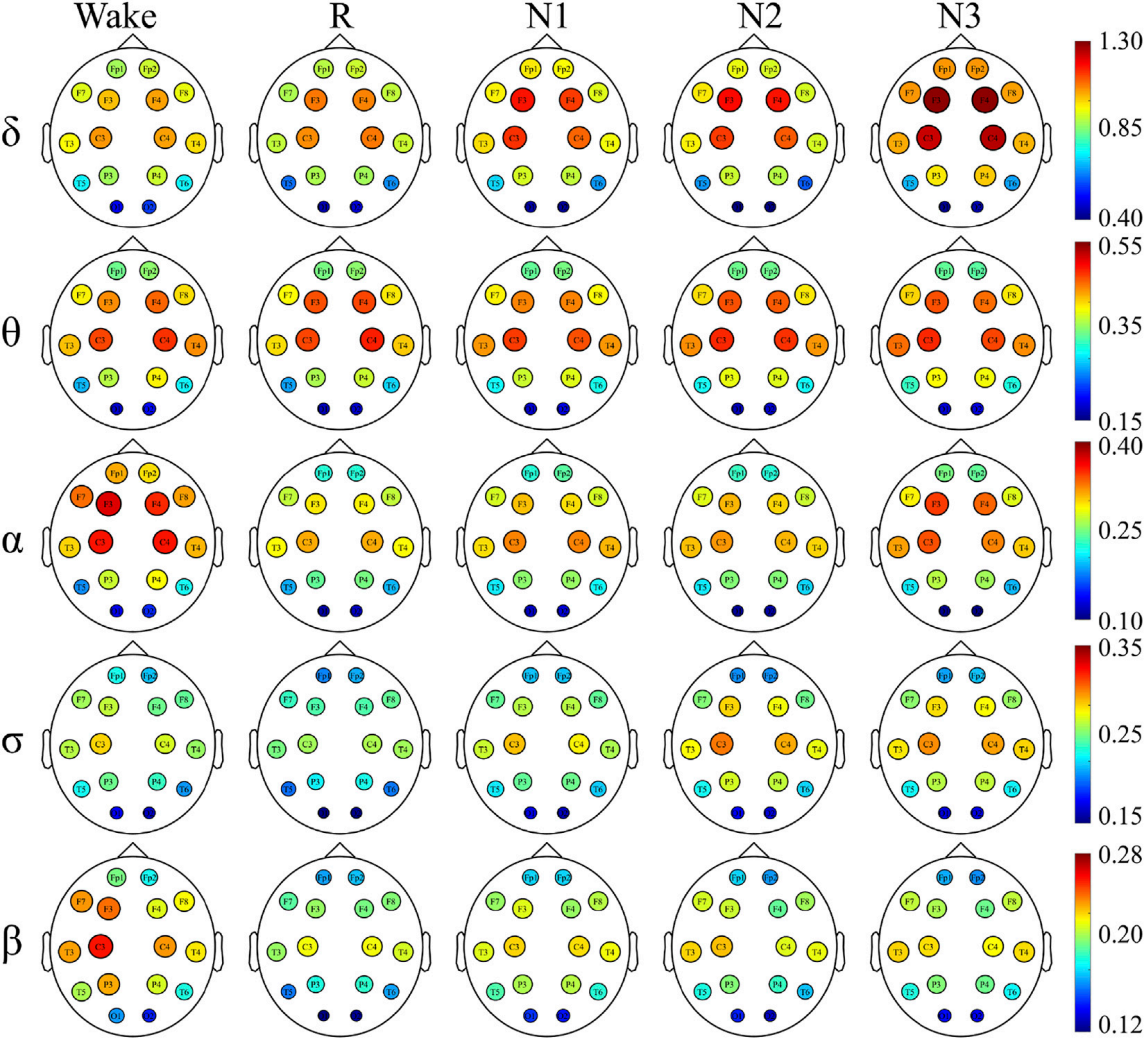
Node strength of the control group.

To have a better understanding of the network hubs, the node strength was studied to analyze the differences between the two groups in the network link weight. Figure 4 and Figure 5 illustrate the average node strength by color and volume of every electrode, respectively. Compared with the control group, the depressed patients were significantly higher in the node strength. All the comparisons of the node strength between the patients and controls showed significant differences.

Notably, the point at which the highest value appears varied with the sleep stage and frequency component. In both the depressed and control groups, the highest node strength was recorded in the N3 stage for δ and σ bands, during W for α and β bands and in the N2 stage for θ band.

There was no significant difference in the dominance between the depressed and control groups. In almost all the sleep stages and frequency bands, the node strength of the central node was significantly higher than that of the other nodes.

### Undirected Connectivity Graphs

**FIGURE 6:**
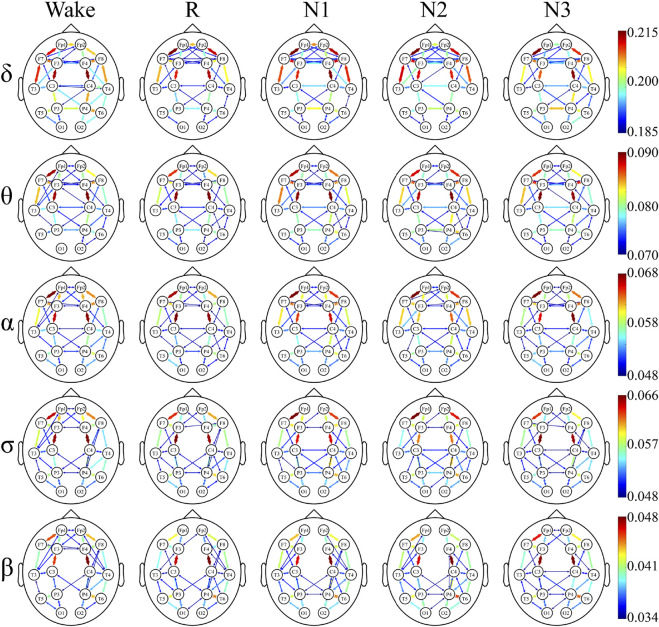
Undirected connectivity graphs of the depressed group. Red indicates a higher link value. No link indicates that there is no direct functional connection between the two sites.

**FIGURE 7:**
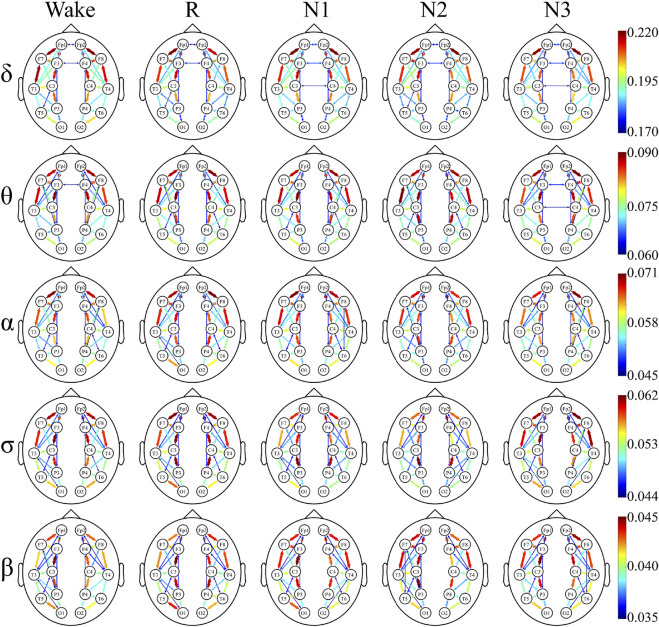
Undirected connectivity graphs of the control group. Red indicates a higher link value. No link indicates that there is no direct functional connection between the two sites.

The undirected connectivity graphs characterized significant changes in the WPLI between the depressed and control groups (Figures 6, 7). The weight of each link was the average of weights of that link in the networks of all the participants in every sleep stage. These figures showed the top 30% connectivity in both the groups of each band and each sleep stage.

The link patterns in the two groups were also different. In the patient group, there were links between the hemispheres in each frequency band and sleep stage. On the contrary, the link of the control group is only observed within each hemisphere, except for each sleep stage in δ and Wake in θ. But there are many long-range links inside the hemisphere in the control group.

Furthermore, in most frequency bands and sleep stages, the anterior cortex was obviously denser than the posterior cortex in the depressed patients. For the control group, this anterior–posterior difference was attenuated, and no clear anterior dominance was noted in the high-frequency bands (σ and β bands).

### Lateralization of Network

The asymmetry of the cortical network between the hemispheres was studied for the depressed and control groups, respectively. A paired t-test was performed to analyze the node strength between the left and right hemisphere belonging to the same sleep stage. The average of node strength and significant differences between the hemispheres are indicated in Table 2. No significant differences for each frequency band and sleep stage were observed in the control group. In the patients group also, no significant differences were noted except for the δ band, where the node strength of the patients’ right hemisphere was significantly higher than that of the left hemisphere in the W, N2, and R stage.

**TABLE 2:** Average node strength in the left and right hemispheres in the δ band for the depressed and control groups. Since other frequency bands did not demonstrate differential properties between the left and right hemispheres, only the result of the δ band is shown.

		W	R	N1	N2	N3
C	L	2.24 ± 0.39	2.13 ± 0.36	2.19 ± 0.40	2.17 ± 0.38	2.29 ± 0.41
	R	2.25 ± 0.38	2.14 ± 0.36	2.20 ± 0.37	2.16 ± 0.37	2.26 ± 0.41
D	L	2.44 ± 0.44*	2.38 ± 0.45*	2.38 ± 0.49	2.38 ± 0.42*	2.54 ± 0.49
	R	2.49 ± 0.45*	2.42 ± 0.44*	2.39 ± 0.50	2.46 ± 0.47*	2.54 ± 0.50

Bold numbers and * indicate significant difference (*p* < 0.05).

L, left hemisphere; R, right hemisphere; C, control group; D, depressed group.

## Discussion

In this study, the differences in the sleep-state functional network topology of the depressed patients were analyzed based on the weighted undirected graph, to provide detailed evidence and potential biomarkers for the abnormal cortical connectivity in depression.

This study showed that the patients had higher global efficiency during sleep. GE reflects the balanced state of global integration in the brain network. GE can reflect the balance of functional segregation and integration (Zhang et al., 2011). The significantly higher GE value in the depressed patients may indicate the abnormal balance of functional segregation and integration of the cortical networks during sleep. Therefore, excessive GE is considered to be a sign of disordered brain function in the depressed patients (Zhang et al., 2011; Zhang et al., 2018). This abnormal change was said to indicate more random brain network structure in the depressed patients (Latora and Marchiori, 2001). Similarly, other studies reported an increased randomness in the brain networks of depressed patients (Hasanzadeh et al., 2017; Li et al., 2017). It can be seen from the calculation formula that GE is related to the reciprocal of the network characteristic path length (CPL) (Latora and Marchiori, 2001). Consequently, the lower CPL of patients reported in previous research is consistent with the results of our research (Hasanzadeh et al., 2017). This randomized structure is thought to be related to the cognitive capacity and abnormalities in the brain functional networks (Latora and Marchiori, 2001; Leistedt et al., 2009). Increased randomization has also been found in fMRI studies (Zhang et al., 2011; Li et al., 2017). Therefore, it is considered that GE can be an important tool for depression screening, and its exploration of differential diagnosis of depression requires further research.

The results indicated that the strength of most nodes in depressed patients was significantly stronger than that in healthy participants, but no change in the node center. The right hemisphere lateralization was found in δ in patients during W, N2, and R stages. Previous studies have pointed out that the dysfunction of the right hemisphere in the depressed patients seems to be an important cause of abnormal information processing (Flor-Henry, 1976; Hou et al., 2016). It has also been reported that patients had less gray matter volume in the right hemisphere and greater gray matter volume in the left hemisphere than the healthy controls (Peng et al., 2019). Our previous research also found some evidence of cortical lateralization in patients (Lian et al., 2021). In addition, transcranial magnetic stimulation over the separate hemisphere can alleviate the imbalance between the hemispheres and effectively improve the patients’ core depression factors and anxiety symptoms (O'Reardon et al., 2007; Carpenter et al., 2017; Cristancho et al., 2019; Li C.-T. et al., 2020; Wang et al., 2022). The effectiveness of this treatment should be related to lateralization in patients. Therefore, the right hemisphere lateralization of the functional network may be caused by the right hemisphere dysfunction in patients.

The distinct patterns of cortical connectivity between the depressed and control group were observed. More links and random structures emerged between the patients’ left and right hemispheres. This abnormal link strength may be related to excessive brain network activation in patients processing information (Rotenberg, 2004). Multiple studies have pointed out significant increases in the brain connections in patients (Greicius et al., 2007; Goodman et al., 2021; Yang et al., 2021). In addition, depression is also considered as a “disconnection syndrome” (Greening et al., 2014; Chen et al., 2016). The depressed group exhibited more interhemispheric connectivity and decreased long-range connectivity. The enhanced functional connectivity between the hemispheres may be due to disruption of the integrity of the corpus callosum (Chen et al., 2016), leading to an unbalanced hemispheric functional coordination. Furthermore, depressed patients had decreased matter volume in the left precentral gyrus and increased gray matter volume in the right thalamus. Abnormal gray matter volume and connectivity indicated abnormal intrinsic wiring costs of the brain structures, resulting in abnormal topological properties of functional connectivity (Zhang et al., 2012; Zhang et al., 2019). The elevated cortisol levels in the depressed patients caused decreased engagement of multiple brain regions, such as the precuneus, medial frontal lobe, posterior cingulate cortex, and inferior temporal gyrus (Peters et al., 2016). These may be possible reasons for the decrease in long-range links during sleep in depressed patients.

Meanwhile, the abnormal cortical network structure in depressed patients may be related to the anterior cingulate cortex and the medial temporal lobe. In our results, the region around the central lead has higher node strength and more number of links than other regions. Related research has suggested that enhanced functional connectivity in the anterior cingulate cortex and medial temporal lobe is associated with depression (de Kwaasteniet et al., 2013). In the past few decades, researchers have studied depression with the EEG signals (Sun et al., 2019; Li X. et al., 2020; Hasanzadeh et al., 2020; Olejarczyk et al., 2020; Olejarczyk et al., 2021; Liu et al., 2022). Increased randomness in the brains of depressed patients has been proven in resting-state EEG studies. Studies using other features, such as the characteristic path length and clustering coefficient, also indicated that the brain functional networks in the depression group were deviated compared to the healthy controls. This study focused on sleep-state EEG because we believe sleep-state EEG is less affected by the subjective factors. We can more clearly describe the sleep-state EEG network in depressed patients using the graph theory from the aspects of connection strength, brain region difference, and connection pattern. An enhanced link between the left and right hemispheres of patients explains increased brain network randomness in depression proposed in previous studies.

Previous studies have suggested that the severity of depression and the presence of other concomitant symptoms affect cortical functional connectivity (Cerullo et al., 2014; Liu et al., 2021). Further research should be carried out in the depressed patients of varying severity and other concomitant symptoms. In addition, age and gender will also be important factors affecting the cortical EEG (Mann and Roschke, 2004; Campos-Beltran and Marshall, 2021; Hong et al., 2021; Putilov et al., 2021). Although we ensured that there were no significant gender differences between the depressed and control groups in this study, due to the small sample size, more participants should be recruited to reduce the gender impact. We anticipated to enroll the patients with mild and moderate depression in further study and explore the performance of network metrics in different depressed patient groups. We expected that the analysis of performance of network metrics can provide a quantitative reference for clinical research and can be used for the screening of depressive disorder and severity of depression.

In summary, this study showed that depressed patients displayed a random cortical functional network and right lateralization in the δ band during sleep. It also revealed detailed differences in the sleep-state functional network topology between the depressed patients and healthy participants, which may contribute to the understanding of the neural mechanisms of depression pathology, as well as the diagnosis and treatment of depression. This study indicated that functional network topology can clearly describe the differences in depressed patients, for which it was thought to be a powerful tool for the physiology and pathology of mental diseases.

## Data Availability

The original contributions presented in the study are included in the article/Supplementary Material. Further inquiries can be directed to the corresponding authors.
